# 9-(Methyl­sulfan­yl)acridinium trifluoro­methane­sulfonate

**DOI:** 10.1107/S1600536809004978

**Published:** 2009-02-21

**Authors:** Beata Zadykowicz, Damian Trzybiński, Artur Sikorski, Jerzy Błażejowski

**Affiliations:** a Faculty of Chemistry, University of Gdańsk, J. Sobieskiego 18, 80-952 Gdańsk, Poland

## Abstract

In the crystal structure of the title compound, C_14_H_12_NS^+^·CF_3_SO_3_^−^, N—H⋯O hydrogen bonds link cations and anions into ion pairs. Inversely oriented ion pairs form stacks through multidirectional π–π inter­actions among the acridine units. The crystal structure features a network of C—H⋯O inter­actions among stacks and also long-range electrostatic inter­actions among ions. In the packing of the mol­ecules, the acridine units are nearly parallel in stacks or inclined at an angle of 33.07 (2)° in the four adjacent stacks with which they inter­act *via* weak C—H⋯O inter­actions. The methyl­sulfanyl group is twisted through an angle of 60.53 (2)° with respect to the acridine ring.

## Related literature

For general background, see: Wróblewska *et al.* (2004 ▶); Zomer & Jacquemijns (2001 ▶). For related structures, see: Meszko *et al.* (2002 ▶); Mrozek *et al.* (2002 ▶); Storoniak *et al.* (2000 ▶). For mol­ecular inter­actions, see: Aakeröy *et al.* (1992 ▶); Bianchi *et al.* (2004 ▶); Hunter *et al.* (2001 ▶); Spek (2009 ▶); Steiner (1991 ▶). For the synthesis, see: Berny *et al.* (1992 ▶); Sato (1996 ▶).
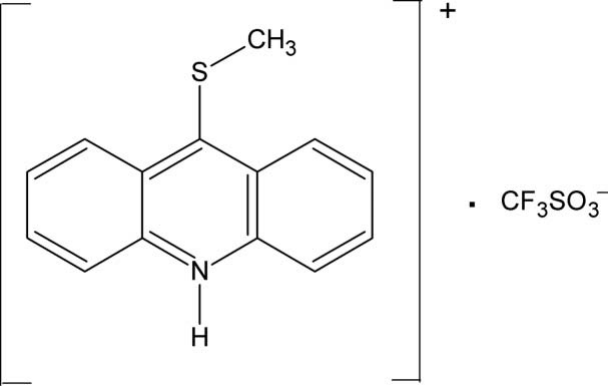


## Experimental

### 

#### Crystal data

C_14_H_12_NS^+^·CF_3_SO_3_^−^
*M* *_r_* = 375.40
Monoclinic, 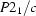

*a* = 7.2992 (2) Å
*b* = 17.3090 (6) Å
*c* = 13.0582 (4) Å
β = 103.910 (3)°
*V* = 1601.42 (9) Å^3^
*Z* = 4
Mo *K*α radiation
μ = 0.38 mm^−1^
*T* = 295 K
0.45 × 0.40 × 0.20 mm

#### Data collection

Oxford Diffraction Gemini R Ultra Ruby CCD diffractometer
Absorption correction: none
13308 measured reflections
2841 independent reflections
1944 reflections with *I* > 2σ(*I*)
*R*_int_ = 0.028

#### Refinement

*R*[*F* ^2^ > 2σ(*F* ^2^)] = 0.050
*wR*(*F* ^2^) = 0.161
*S* = 1.06
2841 reflections
226 parameters
1 restraint
H atoms treated by a mixture of independent and constrained refinement
Δρ_max_ = 0.51 e Å^−3^
Δρ_min_ = −0.35 e Å^−3^

### 

Data collection: *CrysAlis CCD* (Oxford Diffraction, 2008 ▶); cell refinement: *CrysAlis RED* (Oxford Diffraction, 2008 ▶); data reduction: *CrysAlis RED*; program(s) used to solve structure: *SHELXS97* (Sheldrick, 2008 ▶); program(s) used to refine structure: *SHELXL97* (Sheldrick, 2008 ▶); molecular graphics: *ORTEPII* (Johnson, 1976 ▶); software used to prepare material for publication: *SHELXL97* and *PLATON* (Spek, 2009 ▶).

## Supplementary Material

Crystal structure: contains datablocks global, I. DOI: 10.1107/S1600536809004978/ng2540sup1.cif

Structure factors: contains datablocks I. DOI: 10.1107/S1600536809004978/ng2540Isup2.hkl

Additional supplementary materials:  crystallographic information; 3D view; checkCIF report

## Figures and Tables

**Table 1:** Hydrogen-bond geometry (Å, °)

*D*—H⋯*A*	*D*—H	H⋯*A*	*D*⋯*A*	*D*—H⋯*A*
C7—H7⋯O18^i^	0.99 (4)	2.38 (4)	3.315 (5)	158 (3)
N10—H10⋯O19	0.86 (2)	1.86 (2)	2.712 (4)	172 (3)

Symmetry code: (i) 
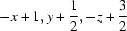
.

**Table 2:** π–π Interactions (Å,°)

*Cg_i_*	*Cg_j_*	*Cg*⋯*Cg*	Dihedral angle	Interplanar distance	Offset
*Cg*1	*Cg*1^ii^	3.827 (2)	0.0	3.468 (2)	1.618 (2)
*Cg*1	*Cg*3^ii^	3.634 (2)	1.4	3.474 (2)	1.066 (2)
*Cg*1	*Cg*3^iii^	3.810 (2)	1.4	3.412 (2)	1.695 (2)
*Cg*2	*Cg*3^ii^	3.830 (2)	4.0	3.492 (2)	1.573 (2)
*Cg*3	*Cg*1^ii^	3.634 (2)	1.4	3.483 (2)	1.037 (2)
*Cg*3	*Cg*1^iii^	3.810 (2)	1.4	3.386 (2)	1.747 (2)
*Cg*3	*Cg*2^ii^	3.830 (2)	4.0	3.449 (2)	1.665 (2)

Symmetry codes: (ii) 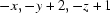; (iii) 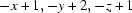. Notes: *Cg*1, *Cg*2 and *Cg*3 are the centroids of the C9/N10/C11–C14, C1–C4/C11/C12 and C5–C8/C13/C14 rings, respectively. *Cg*⋯*Cg* is the distance between ring centroids. The dihedral angle is that between the planes of rings *Cg_i_* and *Cg_j_*. The interplanar distance is the perpendicular distance of *Cg_i_* from ring *j*. The offset is the perpendicular distance of ring *i* from ring *j*.

**Fig. 1.**
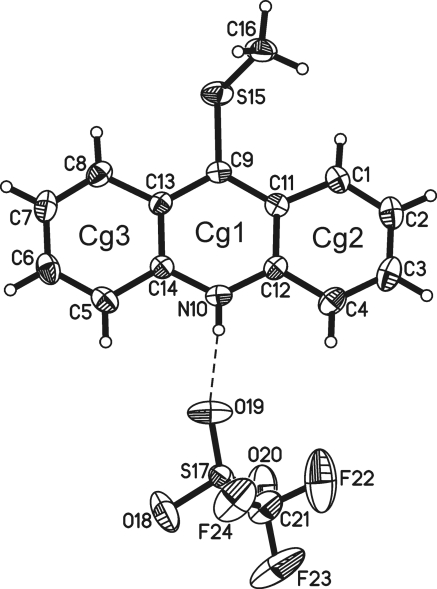
The molecular structure of the title compound showing the atom-labeling scheme. Displacement ellipsoids are drawn at the 25% probability level and H atoms are shown as small spheres of arbitrary radius. The N10–H10···O19 hydrogen bond is represented by a dashed line. Cg1, Cg2 and Cg3 denote the ring centroids.

**Fig. 2.**
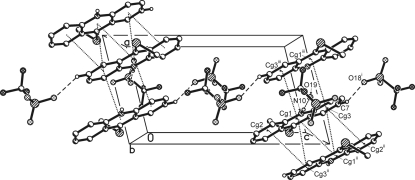
The arrangement of the ions in the crystal structure. The N–H···O and C–H···O hydrogen bonds are represented by dashed lines, the π-π contacts by dotted lines. H atoms not involved in the interactions have been omitted. [Symmetry codes: (i) -x + 1, y + 1/2, -z + 3/2; (ii) -x, -y + 2, -z + 1; (iii) -x + 1, -y + 2, -z + 1.]

**Fig. 3.**
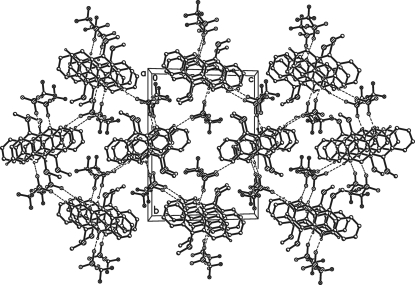
Stacks of the ion pairs in the crystal structure viewed along the a axis. The N–H···O and C–H···O interactions are represented by dashed lines. H atoms not involved in interactions have been omitted.

## supplementary crystallographic information

### Comment

Acridinium cations containing various substituents at position 9 and alkyl substitutes at the endocyclic N atom (position 10) are susceptible to oxidation by H_2_O_2_ or other oxidants in alkaline media, leading to the formation of electronically excited 10-alkyl-9-acridinones capable of emitting light with a quantum yield of several percent (Zomer & Jacquemijns, 2001; Wróblewska *et al.*, 2004). The chemiluminescence phenomenon described above is governed by the features of the substituent at position 9. In the search for derivatives that could exhibit enhanced chemiluminescence, we turned our attention to compounds in which the C atom at position 9 is bound to the S atom. The simplest compound that we were able to synthesize was 9-(methylthio)acridinium trifluoromethanesulfonate. It was obtained by the reaction of 9-thioacridinone (Berny *et al.*, 1992) with methyl trifluoromethanesulfonate, which usually leads to quarternarization of the endocyclic N atom (Sato, 1996). The cation of the reaction product has a protonated endocyclic N atom, enabling it to react with oxidants, thereby facilitating the investigation of chemiluminescence phenomena. This paper presents the crystal structure of the title compound. This is, to our knowledge, only the second report on the crystal structure of an acridine derivatives S-substitued at position 9 (for the first one, see Mrozek *et al.*, 2002).

In the cations of the title compound (Fig. 1), the bond lenghts and angles characterizing the geometry of the acridine skeleton are typical of acridine-based derivatives (Storoniak *et al.*, 2000; Meszko *et al.*, 2002; Mrozek *et al.*, 2002). The C9–S15 and S15–C16 bond lengths (1.754 (3) Å and 1.807 (4) Å, respectively) correlate well with those reported for 9-(thio-2'-methyl-4'-nitrophenyl)acridine (Mrozek *et al.*, 2002). The C9–S15–C16 fragment and the acridine ring system, with an average deviation from planarity of 0.037 (4) Å, are oriented at 60.53 (2)° to each other. The acridine units in the lattice are either parallel (within stacks) or inclined at an angle of 33.07 (2)° (in four adjacent stacks with which they interact *via* C–H···O hydrogen bonds).

In the crystal structure, N–H···O hydrogen bonds (Aakeröy *et al.*, 1992) link cations and anions in ion pairs (Table 1, Fig. 1). Inversely oriented ion pairs form stacks in which the central ring (*Cg*1) and the aromatic rings (*Cg*2 and *Cg*3) are involved in multidirectional π-π interactions (Table 2, Fig. 2) of an attractive nature (Hunter *et al.*, 2001). The crystal structure is stabilized by a network of C–H···O hydrogen type bonding interactions (Steiner, 1991; Bianchi *et al.*, 2004) between neighbouring stacks (Figs 2 and 3) as well as by long-range electrostatic interactions between ions.

### Experimental

9-(Methylthio)acridinium trifluoromethanesulfonate was synthesized in two steps. First, 9-thioacridinone was synthesized by heating with stirring a mixture of 9(10*H*)-acridinone, tetraphosphorus decasulfide and freshly distilled pyridine at 100°C for 1 h (Berny *et al.*, 1992). The reactant mixture was subsequently poured into 30% aq ammonia and the resulting precipitate of 9-thioacridinone filtered off. This compound was then treated with a fivefold molar excess of methyl triluoromethanesulfonate dissolved in dichloromethane for 3 h (Ar athmosphere, room temperature) (Sato, 1996). The crude 9-(methylthio)acridinium trifluoromethanesulfonate thus formed was dissolved in a small amount of ethanol, filtered, and again precipitated with a 25 *v*/*v* excess of diethyl ether (yield: 87%). Yellow crystals suitable for X-ray investigations were grown from absolute ethanol solution (m.p. 421–423 K).

### Refinement

H atoms involved in C–H···O interactions were located in a difference map and refined without constrains. H atoms involved in N–H···O interaction were located in a difference map and refined using the N—H distance restraint of 0.86 (2) Å. Other H atoms were positioned geometrically, with C—H = 0.93 Å (aromatic) and 0.96 Å (methyl), and constrained to ride on their parent atoms with *U*_iso_(H) = 1.2*U*_eq_(C) (aromatic) or *U*_iso_(H) = 1.5*U*_eq_(C) (methyl).

### Crystal data

**Table:**

C_14_H_12_NS^+^·CF_3_SO_3_^−^	*F*(000) = 768
*M**_r_* = 375.40	*D*_x_ = 1.557 Mg m^−^^3^
Monoclinic, *P*2_1_/*c*	Mo *K*α radiation, λ = 0.71073 Å
Hall symbol: -P 2ybc	Cell parameters from 5491 reflections
*a* = 7.2992 (2) Å	θ = 3.1–29.2°
*b* = 17.3090 (6) Å	µ = 0.38 mm^−^^1^
*c* = 13.0582 (4) Å	*T* = 295 K
β = 103.910 (3)°	Block, yellow
*V* = 1601.42 (9) Å^3^	0.45 × 0.4 × 0.2 mm
*Z* = 4	

### Data collection

**Table:**

Oxford Diffraction Gemini R Ultra Ruby CCD diffractometer	1944 reflections with *I* > 2σ(*I*)
Radiation source: Enhance (Mo) X-ray Source	*R*_int_ = 0.028
graphite	θ_max_ = 25.1°, θ_min_ = 3.1°
Detector resolution: 10.4002 pixels mm^-1^	*h* = −8→8
ω scans	*k* = −20→20
13308 measured reflections	*l* = −15→15
2841 independent reflections	

### Refinement

**Table:**

Refinement on *F*^2^	Primary atom site location: structure-invariant direct methods
Least-squares matrix: full	Secondary atom site location: difference Fourier map
*R*[*F*^2^ > 2σ(*F*^2^)] = 0.050	Hydrogen site location: inferred from neighbouring sites
*wR*(*F*^2^) = 0.161	H atoms treated by a mixture of independent and constrained refinement
*S* = 1.06	*w* = 1/[σ^2^(*F*_o_^2^) + (0.1054*P*)^2^] where *P* = (*F*_o_^2^ + 2*F*_c_^2^)/3
2841 reflections	(Δ/σ)_max_ = 0.001
226 parameters	Δρ_max_ = 0.51 e Å^−^^3^
1 restraint	Δρ_min_ = −0.35 e Å^−^^3^

### Special details

**Table:**

Geometry. All e.s.d.'s (except the e.s.d. in the dihedral angle between two l.s. planes) are estimated using the full covariance matrix. The cell e.s.d.'s are taken into account individually in the estimation of e.s.d.'s in distances, angles and torsion angles; correlations between e.s.d.'s in cell parameters are only used when they are defined by crystal symmetry. An approximate (isotropic) treatment of cell e.s.d.'s is used for estimating e.s.d.'s involving l.s. planes.

### Fractional atomic coordinates and isotropic or equivalent isotropic displacement parameters (Å^2^)

**Table:**

	*x*	*y*	*z*	*U*_iso_*/*U*_eq_	
C1	−0.0169 (4)	1.03470 (19)	0.2103 (2)	0.0567 (7)	
H1	−0.0740	1.0824	0.1916	0.068*	
C2	−0.0343 (4)	0.9785 (2)	0.1374 (2)	0.0702 (9)	
H2	−0.1039	0.9879	0.0690	0.084*	
C3	0.0508 (5)	0.9058 (2)	0.1626 (3)	0.0751 (10)	
H3	0.0404	0.8684	0.1103	0.090*	
C4	0.1478 (4)	0.8897 (2)	0.2624 (3)	0.0630 (8)	
H4	0.2023	0.8414	0.2791	0.076*	
C5	0.3767 (3)	0.9596 (2)	0.6240 (2)	0.0577 (8)	
H5	0.4190	0.9092	0.6380	0.069*	
C6	0.4067 (4)	1.0138 (2)	0.7023 (2)	0.0657 (9)	
H6	0.4717	0.9998	0.7700	0.079*	
C7	0.3425 (4)	1.0895 (2)	0.6832 (3)	0.0670 (9)	
H7	0.380 (4)	1.127 (2)	0.742 (3)	0.079 (10)*	
C8	0.2485 (4)	1.11202 (18)	0.5862 (2)	0.0573 (7)	
H8	0.2070	1.1628	0.5750	0.069*	
C9	0.1176 (3)	1.07880 (15)	0.3956 (2)	0.0442 (6)	
N10	0.2546 (3)	0.93052 (14)	0.44086 (19)	0.0478 (6)	
H10	0.300 (4)	0.8858 (12)	0.462 (2)	0.071 (10)*	
C11	0.0877 (3)	1.02193 (16)	0.3156 (2)	0.0453 (6)	
C12	0.1652 (3)	0.94706 (16)	0.3405 (2)	0.0455 (6)	
C13	0.2116 (3)	1.05863 (16)	0.49974 (19)	0.0438 (6)	
C14	0.2796 (3)	0.98202 (16)	0.5208 (2)	0.0441 (6)	
S15	0.03215 (12)	1.17329 (5)	0.36941 (8)	0.0731 (3)	
C16	0.1546 (5)	1.2067 (2)	0.2729 (3)	0.0770 (10)	
H16A	0.1099	1.2572	0.2488	0.116*	
H16B	0.1314	1.1716	0.2142	0.116*	
H16C	0.2877	1.2089	0.3044	0.116*	
S17	0.36383 (10)	0.71528 (5)	0.50824 (6)	0.0577 (3)	
O18	0.4183 (4)	0.6911 (2)	0.6130 (2)	0.1215 (12)	
O19	0.4246 (4)	0.79237 (14)	0.4965 (3)	0.1040 (10)	
O20	0.1780 (3)	0.6955 (2)	0.4545 (2)	0.1045 (10)	
C21	0.5109 (6)	0.6611 (3)	0.4413 (4)	0.0960 (13)	
F22	0.4700 (5)	0.6751 (3)	0.3414 (3)	0.193 (2)	
F23	0.4819 (6)	0.5867 (2)	0.4487 (4)	0.2000 (19)	
F24	0.6911 (3)	0.67460 (17)	0.4790 (3)	0.1366 (11)	

### Atomic displacement parameters (Å^2^)

**Table:**

	*U*^11^	*U*^22^	*U*^33^	*U*^12^	*U*^13^	*U*^23^
C1	0.0519 (15)	0.068 (2)	0.0489 (15)	0.0013 (14)	0.0100 (12)	0.0050 (15)
C2	0.0620 (18)	0.096 (3)	0.0504 (17)	−0.0082 (18)	0.0092 (14)	−0.0036 (18)
C3	0.0695 (19)	0.092 (3)	0.067 (2)	−0.0095 (19)	0.0227 (17)	−0.031 (2)
C4	0.0542 (16)	0.059 (2)	0.077 (2)	0.0015 (14)	0.0176 (15)	−0.0158 (16)
C5	0.0438 (14)	0.070 (2)	0.0586 (18)	−0.0061 (13)	0.0101 (13)	0.0162 (16)
C6	0.0537 (16)	0.095 (3)	0.0466 (17)	−0.0186 (17)	0.0077 (13)	0.0052 (17)
C7	0.0646 (18)	0.086 (3)	0.0510 (18)	−0.0206 (18)	0.0156 (15)	−0.0155 (18)
C8	0.0582 (16)	0.0533 (19)	0.0639 (19)	−0.0093 (13)	0.0218 (14)	−0.0097 (14)
C9	0.0383 (12)	0.0407 (16)	0.0555 (16)	0.0005 (11)	0.0151 (11)	0.0019 (12)
N10	0.0417 (11)	0.0395 (14)	0.0618 (15)	0.0004 (10)	0.0118 (10)	0.0048 (12)
C11	0.0379 (12)	0.0494 (17)	0.0499 (15)	0.0018 (11)	0.0129 (11)	0.0016 (12)
C12	0.0375 (12)	0.0475 (17)	0.0534 (16)	−0.0055 (11)	0.0144 (11)	−0.0059 (13)
C13	0.0400 (12)	0.0448 (16)	0.0496 (15)	−0.0082 (11)	0.0166 (11)	−0.0039 (12)
C14	0.0353 (12)	0.0488 (17)	0.0489 (15)	−0.0069 (11)	0.0113 (11)	0.0023 (12)
S15	0.0800 (6)	0.0507 (6)	0.0909 (7)	0.0178 (4)	0.0248 (5)	0.0059 (4)
C16	0.083 (2)	0.056 (2)	0.087 (2)	−0.0126 (17)	0.0096 (19)	0.0185 (17)
S17	0.0620 (5)	0.0550 (5)	0.0527 (5)	−0.0016 (3)	0.0070 (3)	0.0084 (3)
O18	0.124 (2)	0.173 (3)	0.0551 (15)	−0.023 (2)	−0.0018 (15)	0.0306 (16)
O19	0.1003 (18)	0.0507 (16)	0.172 (3)	0.0158 (13)	0.0536 (18)	0.0300 (16)
O20	0.0605 (14)	0.159 (3)	0.0864 (18)	−0.0124 (15)	0.0026 (13)	−0.0175 (17)
C21	0.077 (3)	0.081 (3)	0.122 (4)	0.005 (2)	0.007 (2)	−0.023 (3)
F22	0.144 (3)	0.345 (6)	0.097 (2)	0.028 (3)	0.044 (2)	−0.063 (3)
F23	0.177 (3)	0.085 (2)	0.331 (6)	−0.001 (2)	0.047 (3)	−0.088 (3)
F24	0.0666 (14)	0.132 (2)	0.199 (3)	0.0187 (13)	0.0096 (15)	−0.057 (2)

### Geometric parameters (Å, °)

**Table:**

C1—C2	1.346 (4)	C9—C11	1.414 (4)
C1—C11	1.421 (4)	C9—S15	1.754 (3)
C1—H1	0.9300	N10—C12	1.347 (3)
C2—C3	1.407 (5)	N10—C14	1.351 (3)
C2—H2	0.9300	N10—H10	0.860 (18)
C3—C4	1.354 (4)	C11—C12	1.420 (4)
C3—H3	0.9300	C13—C14	1.419 (4)
C4—C12	1.407 (4)	S15—C16	1.807 (4)
C4—H4	0.9300	C16—H16A	0.9600
C5—C6	1.365 (5)	C16—H16B	0.9600
C5—C14	1.417 (4)	C16—H16C	0.9600
C5—H5	0.9300	S17—O18	1.393 (3)
C6—C7	1.394 (5)	S17—O20	1.411 (2)
C6—H6	0.9300	S17—O19	1.426 (3)
C7—C8	1.346 (4)	S17—C21	1.803 (5)
C7—H7	1.00 (4)	C21—F22	1.289 (6)
C8—C13	1.433 (4)	C21—F23	1.312 (5)
C8—H8	0.9300	C21—F24	1.310 (5)
C9—C13	1.412 (4)		
			
C2—C1—C11	120.6 (3)	C9—C11—C1	124.0 (3)
C2—C1—H1	119.7	C12—C11—C1	117.1 (2)
C11—C1—H1	119.7	N10—C12—C4	119.6 (3)
C1—C2—C3	121.4 (3)	N10—C12—C11	119.4 (2)
C1—C2—H2	119.3	C4—C12—C11	121.1 (3)
C3—C2—H2	119.3	C9—C13—C14	119.0 (2)
C4—C3—C2	120.5 (3)	C9—C13—C8	123.7 (3)
C4—C3—H3	119.7	C14—C13—C8	117.3 (2)
C2—C3—H3	119.7	N10—C14—C13	119.4 (2)
C3—C4—C12	119.2 (3)	N10—C14—C5	119.9 (3)
C3—C4—H4	120.4	C13—C14—C5	120.8 (3)
C12—C4—H4	120.4	C9—S15—C16	102.83 (15)
C6—C5—C14	118.5 (3)	S15—C16—H16A	109.5
C6—C5—H5	120.7	S15—C16—H16B	109.5
C14—C5—H5	120.7	H16A—C16—H16B	109.5
C5—C6—C7	121.7 (3)	S15—C16—H16C	109.5
C5—C6—H6	119.2	H16A—C16—H16C	109.5
C7—C6—H6	119.2	H16B—C16—H16C	109.5
C8—C7—C6	120.9 (3)	O18—S17—O20	115.38 (19)
C8—C7—H7	121 (2)	O18—S17—O19	111.4 (2)
C6—C7—H7	117 (2)	O20—S17—O19	117.40 (19)
C7—C8—C13	120.8 (3)	O18—S17—C21	104.9 (2)
C7—C8—H8	119.6	O20—S17—C21	104.20 (19)
C13—C8—H8	119.6	O19—S17—C21	101.4 (2)
C13—C9—C11	119.5 (2)	F22—C21—F23	105.0 (5)
C13—C9—S15	119.1 (2)	F22—C21—F24	108.3 (4)
C11—C9—S15	121.4 (2)	F23—C21—F24	108.2 (4)
C12—N10—C14	123.7 (2)	F22—C21—S17	111.9 (3)
C12—N10—H10	124 (2)	F23—C21—S17	110.5 (4)
C14—N10—H10	112 (2)	F24—C21—S17	112.7 (3)
C9—C11—C12	118.9 (2)		
			
C11—C1—C2—C3	−0.4 (5)	S15—C9—C13—C8	−2.4 (3)
C1—C2—C3—C4	2.2 (5)	C7—C8—C13—C9	−178.6 (2)
C2—C3—C4—C12	−1.0 (5)	C7—C8—C13—C14	−0.6 (4)
C14—C5—C6—C7	0.8 (4)	C12—N10—C14—C13	−1.0 (4)
C5—C6—C7—C8	−0.2 (5)	C12—N10—C14—C5	−179.6 (2)
C6—C7—C8—C13	0.1 (4)	C9—C13—C14—N10	0.7 (3)
C13—C9—C11—C12	−4.4 (3)	C8—C13—C14—N10	−177.4 (2)
S15—C9—C11—C12	178.03 (18)	C9—C13—C14—C5	179.3 (2)
C13—C9—C11—C1	175.3 (2)	C8—C13—C14—C5	1.2 (3)
S15—C9—C11—C1	−2.3 (3)	C6—C5—C14—N10	177.3 (2)
C2—C1—C11—C9	177.8 (3)	C6—C5—C14—C13	−1.3 (4)
C2—C1—C11—C12	−2.5 (4)	C13—C9—S15—C16	120.7 (2)
C14—N10—C12—C4	179.4 (2)	C11—C9—S15—C16	−61.7 (2)
C14—N10—C12—C11	−1.4 (4)	O18—S17—C21—F22	177.1 (4)
C3—C4—C12—N10	177.3 (3)	O20—S17—C21—F22	55.5 (4)
C3—C4—C12—C11	−1.9 (4)	O19—S17—C21—F22	−66.9 (4)
C9—C11—C12—N10	4.2 (4)	O18—S17—C21—F23	60.6 (4)
C1—C11—C12—N10	−175.6 (2)	O20—S17—C21—F23	−61.0 (4)
C9—C11—C12—C4	−176.7 (2)	O19—S17—C21—F23	176.6 (4)
C1—C11—C12—C4	3.6 (4)	O18—S17—C21—F24	−60.6 (4)
C11—C9—C13—C14	2.0 (3)	O20—S17—C21—F24	177.8 (4)
S15—C9—C13—C14	179.63 (17)	O19—S17—C21—F24	55.4 (4)
C11—C9—C13—C8	−180.0 (2)		

### Hydrogen-bond geometry (Å, °)

**Table:**

*D*—H···*A*	*D*—H	H···*A*	*D*···*A*	*D*—H···*A*
C7—H7···O18^i^	0.99 (4)	2.38 (4)	3.315 (5)	158 (3)
N10—H10···O19	0.86 (2)	1.86 (2)	2.712 (4)	172 (3)

Symmetry codes:  (i) −*x*+1, *y*+1/2, −*z*+3/2.

### Table 2 π–π Interactions (Å,°).

**Table:**

*Cg_I_*	*Cg_J_*	*Cg*···*Cg*	Dihedral angle	Interplanar distance	Offset
*Cg*1	*Cg*1^ii^	3.827 (2)	0.0	3.468 (2)	1.618 (2)
*Cg*1	*Cg*3^ii^	3.634 (2)	1.44	3.474 (2)	1.066 (2)
*Cg*1	*Cg*3^iii^	3.810 (2)	1.44	3.412 (2)	1.695 (2)
*Cg*2	*Cg*3^ii^	3.830 (2)	3.96	3.492 (2)	1.573 (2)
*Cg*3	*Cg*1^ii^	3.634 (2)	1.44	3.483 (2)	1.037 (2)
*Cg*3	*Cg*1^iii^	3.810 (2)	1.44	3.386 (2)	1.747 (2)
*Cg*3	*Cg*2^ii^	3.830 (2)	3.96	3.449 (2)	1.665 (2)

Symmetry codes: (ii) -x, -y+2, -z+1; (iii) -x+1, -y+2, -z+1. Notes: Cg1, Cg2 and Cg3 are the centroids of the C9/N10/C11–C14, C1–C4/C11/C12 and C5–C8/C13/C14 rings, respectively. Cg···Cg is the distance between ring centroids. The dihedral angle is that between the planes of the rings *Cg_I_* and *Cg_J_*. The interplanar distance is the perpendicular distance of *Cg_I_* from ring *J*. The offset is the perpendicular distance of ring *I* from ring *J*.
